# Evaluation of the Biological Potential of an Endophytic *Fusarium equiseti* from *Pancratium maritimum* Supported by Metabolic Profiling and Molecular Docking

**DOI:** 10.3390/microorganisms14081773

**Published:** 2026-08-12

**Authors:** Amal A. Al Mousa, Mohamed E. Abouelela, Reda A. Abdelhamid, Hassan Mohamed, Mohammed M. M. Abdelrahem, Mohamed F. Awad, Shurouq A. Fahmy, Huda A. Aljhne, Nageh F. Abo-Dahab, Abdallah M. A. Hassane

**Affiliations:** 1 Department of Botany and Microbiology, College of Science, King Saud University, P.O. Box 145111, Riyadh ZIP 4545, Saudi Arabia; 445205534@student.ksu.edu.sa; 2 Department of Pharmacognosy, Faculty of Pharmacy (Boys), Al-Azhar University, Cairo 11884, Egypt; m_abouelela@azhar.edu.eg; 3 Department of Pharmacognosy, Faculty of Pharmacy (Boys), Al-Azhar University, Assiut Branch, Assiut 71524, Egypt; reda.ahmed@azhar.edu.eg; 4 Department of Botany and Microbiology, Faculty of Science, Al-Azhar University, Assiut Branch, Assiut 71524, Egypt; hassanmohamed85@azhar.edu.eg (H.M.); mohammedabdel-rahem.42@azhar.edu.eg (M.M.M.A.); m.fadl@tu.edu.sa (M.F.A.); abodahabn@azhar.edu.eg (N.F.A.-D.); 5 Colin Ratledge Center for Microbial Lipids, School of Agricultural Engineering and Food Science, Shandong University of Technology, Zibo 255000, China; 6 Ministry of Health and Populations, Cairo P.O. Box 11516, Egypt; shorouk.ali@msa.edu.eg

**Keywords:** *Fusarium equiseti*, *Pancratium maritimum* L., LC-MS/MS, Anti-inflammatory, inducible nitric oxide synthase, molecular docking

## Abstract

Endophytic fungi are promising sources of chemically diverse metabolites. Fusarium species exhibit diverse biological roles, ranging from beneficial applications to detrimental effects, including plant diseases and mycotoxin production. A few reports have investigated *Fusarium equiseti* bioactivity, but no reports have evaluated its anti-inflammatory potential. In the present study, the ethyl acetate (EtOAc) extract obtained from an endophytic *F. equiseti*, isolated for the first time from the bulb of *Pancratium maritimum* L., was evaluated for its in vitro antioxidant, cytotoxic, anti-acetyl- and butyrylcholinesterases, and anti-inflammatory activities. The extract was chemically profiled by LC-MS/MS, evaluated in complementary antioxidant and cytotoxicity screens, and assessed for inhibition of nitric oxide (NO) production in lipopolysaccharide-stimulated RAW264.7 macrophages. In addition, putatively annotated metabolites were further examined by molecular docking against inducible nitric oxide synthase (iNOS), as a promising biological activity providing a target directly aligned with the NO-based assay. The EtOAc extract showed total phenolics content of 29.31 mg GAE/g and a total alkaloid content of 8.43 mg AE/g, with a DPPH radical scavenging IC_50_ value of 2.18 mg/mL. Cytotoxicity assessment against the RAW264.7 cell line revealed an IC_50_ value of 49.95 μg/mL, while the *Artemia salina* lethality assay yielded an LC_50_ value of 1.86 mg/mL. Notably, the extract demonstrated potent anti-inflammatory activity through nitric oxide inhibition, with an IC_50_ value of 4.70 μg/mL compared with 18.46 μg/mL for quercetin. LC-MS/MS analysis offered the tentative identification of 31 metabolites in the extract. Docking against iNOS identified fusarioxazin as the highest-scoring ligand (−8.8 kcal/mol), although these computational predictions require experimental validation with purified compounds. The data identify suppression of macrophage NO production as the principal bioactivity of the crude *F. equiseti* TU-64 extract. Collectively, these findings highlight the potential of endophytic *F. equiseti* as a promising source of bioactive metabolites for the development of natural therapeutic agents.

## 1. Introduction

*Pancratium maritimum* L. (Amaryllidaceae) is an indigenous Mediterranean coastal plant recognized as a rich source of structurally diverse secondary metabolites such as Amaryllidaceae alkaloids, including lycorine, galanthamine, pancratistatin, and related alkaloids in addition to phenolic acids, phenylpropanoids, flavonoids, organic acids, and fatty acids [1,2].

Extracts of *P. maritimum* have demonstrated antioxidant, antimicrobial, cytotoxic, antiproliferative, antiviral, antimalarial, and anti-inflammatory-related activities [3,4,5,6]. Ethanolic bulb extracts showed free-radical-scavenging activity, supporting the antioxidant potential of the plant [7]. Earlier investigations also demonstrated dose- and time-dependent antiproliferative effects of *P. maritimum* extracts against MDA-MB-231 breast cancer cells [8]. Notably, pancratistatin isolated from *P. maritimum* has showed potent growth-inhibitory activity against MDA-MB-231, HeLa, and HCT116 cancer cells [1].

Recent evidence further suggests that *P. maritimum* possesses biologically relevant anti-inflammatory and gastroprotective potential. In an ethanol-induced gastric-ulcer model in rats, the plant extract reduced oxidative damage and inflammatory markers, including TNF-α, NLRP3, MyD88, NF-κB, HMGB1, and TLR4-associated responses, while improving gastric tissue morphology [2]. Lycorine, a characteristic alkaloid reported from *P. maritimum*, has demonstrated anti-inflammatory properties in experimental studies [9,10]. Narciclasine, another bioactive compound, has shown particularly pronounced anti-inflammatory effects in preclinical models [11].

Reducing oxidative stress has been consistently linked to anticancer benefits. Cancer is a multifactorial and progressive disease that involves uncontrolled proliferation of abnormal cells, leading to tumor formation and metastasis that can originate in almost any tissue of the body and has the potential to penetrate nearby tissues and disseminate to distant sites [12]. Natural products represent an important reservoir of structurally diverse bioactive compounds for anticancer drug discovery, and several clinically established chemotherapeutic agents have originated from natural sources [13,14]. In this context, extracts of *P. maritimum* obtained from different plant parts and geographical origins have demonstrated promising antiproliferative/anticancer and anti-inflammatory activities, highlighting this species as a potential source of pharmacologically relevant secondary metabolites [15].

Endophytic fungi are endosymbiotic microorganisms that colonize the internal tissues of plants without causing apparent disease symptoms. These fungi contribute to host plant fitness by enhancing tolerance to both biotic and abiotic stresses through various protective mechanisms [16,17]. In addition to their ecological significance, endophytic fungi are recognized as prolific producers of structurally diverse secondary bioactive metabolites. Many of these metabolites are identical or closely related to compounds originally reported from their host plants, a phenomenon that has been attributed to long-term co-evolutionary processes and possible genetic exchange between the host and its associated endophytes [18]. The isolation and characterization of bioactive metabolites from endophytic fungi have attracted considerable scientific interest in recent years due to their pharmaceutical and biotechnological potential. Endophytic fungi have been reported to produce a wide range of secondary metabolites, including phenolic compounds, xanthones, steroids, isocoumarins, alkaloids, terpenoids, and quinones. These metabolites exhibit diverse biological activities, such as anticancer, insecticidal, antiviral, antibacterial, antifungal, anti-inflammatory, antioxidant, and antidiabetic effects [19,20,21].

The genus *Fusarium* (Nectriaceae, Hypocreales) currently includes more than 400 species classified into 23 species complexes [22]. *Fusarium equiseti* (Corda) Sacc. is clustered into clade *Equiseti* of the *F. incarnatum-equiseti* species complex (FIESC) [23] and occurs in diverse terrestrial and aquatic habitats as a phytopathogen, saprotroph, or endophyte. Although some *F. equiseti* strains are associated with plant disease and mycotoxin production [24,25,26,27], others produce structurally diverse secondary metabolites with biological or biotechnological value [28,29,30].

Studies examining endophytic fungi associated with *P. maritimum* remain limited, and, to the best of our knowledge, *F. equiseti* has not previously been reported as an endophyte isolated from the bulbs of this plant. We therefore isolated and characterized an endophytic *F. equiseti* strain (TU-64) from *P. maritimum* and profiled its EtOAc extract by LC–MS/MS. Preliminary biological screening included antioxidant, cytotoxicity, brine shrimp lethality, and cholinesterase assays; however, the principal biological focus was the extract’s anti-inflammatory effect, assessed as inhibition of NO production in LPS-stimulated RAW264.7 macrophages.

## 2. Materials and Methods

### 2.1. Isolation and Molecular Identification of the Fungal Strain

*Fusarium equiseti* (Corda) Sacc. was isolated as an endophytic fungus from healthy bulbs of *P. maritimum* L. [31] according to the method described by Kumar et al. [32]. Endophytic fungi were isolated using potato dextrose agar (PDA) medium (HiMedia, Mumbai, India). The obtained fungal isolate was purified by repeated subculturing and maintained on PDA slants at 4 °C until further use. Molecular identification of the isolated fungus was performed through amplification and sequencing of the internal transcribed spacer (ITS) region of the ribosomal DNA, following the protocol described by Abdelrahem et al. [33]. Genomic DNA was extracted, and PCR amplification was carried out using the universal fungal primers ITS1 (5′-TCC GTA GGT GAA CCT GCG G-3′) and ITS4 (5′-TCC TCC GCT TAT TGA TAT GC-3′). The BioEdit software package No. 7.2.5 was used to analyze and study the ITS sequences. The GenBank BLAST search method (www.ncbi.nlm.nih.gov/BLAST/, accessed on 13 January 2026) was used to perform homology searches by comparing the sequences with reference strains that were available in public sequencing databases.

### 2.2. Fungal Fermentation and Metabolites Extraction

The *F. equiseti* isolate was fermented in sterilized wet rice medium (100 g rice in 110 mL deionized water). After a month of incubation at 28 ± 2 °C, the extract was twice extracted with ethyl acetate (EtOAc) (AL-Nasr Chemicals Co., Cairo, Egypt). The extract was then filtered through anhydrous Na_2_SO_4_ (AL-Nasr Chemicals Co., Cairo, Egypt), evaporated, and stored at −4 °C [34].

### 2.3. Determination of Total Phenolic Content

A modified procedure that was adopted from Suleria et al. [35] was used to determine the total phenolic content. In short, 0.5 mL of the extract solution (10 mg/mL) was combined with an equivalent volume of Folin–Ciocalteu reagent (Alpha Chemika, Mumbai, India), and the mixture was then combined with 1 mL of a 10% sodium carbonate solution (AL-Nasr Chemicals Co., Cairo, Egypt). After that, the mixture was incubated in the dark with constant shaking at 25 °C for 60 min at 180 rpm. A spectrophotometer was used to measure absorbance at 750 nm. Using a gallic acid calibration curve, the total phenolics were computed and represented as gallic acid equivalents (GAE, mg/g) (Alpha Chemika, Mumbai, India) using the formula y = 0.0169x − 0.1172 (R^2^ = 0.9588). Over the level range of 0.5–100 μg/mL, the standard calibration curve showed linearity.

### 2.4. Determination of Total Alkaloids Content

Bromocresol Green (BCG) (Sigma-Aldrich Chemie GmbH, Taufkirchen, Germany) was used to determine the total alkaloids spectrophotometrically [36]. Ten milligrams of EtOAc extract were melted in one milliliter of methanol and combined with five milliliters of BCG reagent that had been adjusted to a pH of 4.7 using phosphate buffer. Five milliliters of chloroform were used to extract the mixture. The separated chloroform layer was collected and its absorbance at 470 nm was measured. Atropine was used to plot the standard curve at values between 10 and 100 μg/mL. Alkaloids were expressed as atropine equivalent (AE) (mg/g) (Alpha Chemika, Mumbai, India) using a standard calibration curve with the formula y = 92.6x + 5.3974.

### 2.5. Antioxidant Activity Assay

The antioxidant activity of the fungal extract was evaluated using the 1,1-diphenyl-2-picrylhydrazyl (DPPH) radical scavenging assay according to a previously described method by Gulcin [37] with slight modifications. DPPH reagent (Sigma-Aldrich, Germany) was prepared as a 0.1 mM solution (0.04 mg/mL) in methanol. Briefly, 0.2 mL of the fungal extract, prepared in 100% methanol at various concentrations ranging from 0.05 to 1.0 mg/mL, was mixed with 1.8 mL of the DPPH solution. A control consisting of DPPH solution and methanol without extract was prepared under the same conditions. The reaction mixtures were incubated in the dark at room temperature for 30 min, after which the absorbance was measured at 517 nm using a UV–Vis spectrophotometer (Jenway 7315, Wolflab Co., York, UK). Butylated hydroxytoluene (BHT) (Chemical Industries Development Company, Cairo, Egypt) was used as a positive control at concentrations ranging from 10 to 100 μg/mL. The percentage of DPPH radical scavenging activity was calculated according to the following equation:

$$\%\;DPPH\;free\;radical\;scavenging\;activity=\frac{A0-A1}{A0}\times 100$$

where *A*_0_ is the absorbance of the negative control (DPPH solution and methanol), and *A*_1_ is the absorbance of the reaction mixture containing DPPH solution, methanol, and the fungal extract. The concentration required to inhibit 50% of the DPPH radicals (IC_50_) was calculated by linear regression analysis using the following equation: IC_50_ = (50 − b)/a where *a* is the slope and *b* is the intercept of the regression equation obtained by plotting the percentage inhibition against the extract concentration.

### 2.6. Acetylcholinesterase and Butyrylcholinesterase Inhibition Assay

See Supplementary Materials.

### 2.7. Brine Shrimp-Based Cytotoxicity Evaluation

The brine shrimp cytotoxicity assay was utilized to determine the fungal extract’s cytotoxic potential against *Artemia salina* larvae. The extract was first dissolved in DMSO and then serially diluted to achieve different quantities. Next, 100 μL of each concentration was mixed with 10 brine shrimp larvae and 5 mL of saltwater. The number of larvae in each treatment that survived after one day of exposure was counted. The percentage mortality was calculated with DMSO as the negative control. Probit analysis was used to calculate the median lethal concentration (IC_50_), which is the concentration that causes 50% larval death [38].

### 2.8. Cytotoxicity Assay Against Mouse Macrophage Cell Line

The murine macrophage cell line RAW 264.7 was obtained from Nawah Scientific Inc. (Mokatam, Cairo, Egypt). Cells were cultured in Dulbecco’s modified Eagle’s medium (DMEM; Gibco, Grand Island, NY, USA) supplemented with 100 U/mL penicillin, 0.1 mg/mL streptomycin (Corning, Glendale, AZ, USA), and 10% heat-inactivated fetal bovine serum (FBS; Gibco, NY, USA). Cells were maintained in a humidified incubator at 37 °C under 5% CO_2_ atmosphere. Cell viability was assessed using the sulforhodamine B (SRB) assay. Briefly, cells were seeded in 96-well plates at a density of 5 × 10^3^ cells/well in 100 μL of complete medium and incubated for 24 h. Thereafter, the medium was replaced with fresh medium containing different concentrations of the tested samples, and cells were incubated for the indicated treatment period. Following treatment, cells were fixed by adding 150 μL of 10% (*w*/*v*) trichloroacetic acid (TCA; Elabscience, Wuhan, China) and incubated at 4 °C for 1 h. The TCA solution was then removed, and cells were washed five times with distilled water. Subsequently, 70 μL of 0.4% (*w*/*v*) sulforhodamine B (SRB; Sigma-Aldrich, Darmstadt, Germany) solution was added to each well, and the plate was incubated in the dark at room temperature for 10 min. Excess dye was removed by washing three times with 1% (*v*/*v*) acetic acid, and the plates were air-dried overnight. Finally, the bound dye was solubilized by adding 150 μL of 10 mM Tris base solution (Elabscience, Wuhan, China), and the absorbance was measured at 540 nm using an Infinite F50 microplate reader (Tecan, Männedorf, Switzerland). Cell viability was calculated relative to untreated controls [39,40].

### 2.9. In Vitro Anti-Inflammatory Assay Against RAW264.7 Cell Line

Murine macrophage RAW 264.7 cells were seeded into 96-well plates and incubated for 24 h. On the following day, inflammation was induced by treatment with 1 μg/mL lipopolysaccharide (LPS), while untreated cells cultured in fresh medium served as the control group. The tested compounds were co-incubated with LPS at five different concentrations (LPS + treatment groups). Quercetin (0.01–100 μg/mL; Sigma-Aldrich Chemie GmbH, Taufkirchen, Germany) was used as a positive anti-inflammatory control. Nitric oxide (NO) production was indirectly determined by measuring nitrite levels in the culture supernatant using the Griess reaction. Briefly, an equal volume of cell culture supernatant and Griess reagent (Sigma-Aldrich, Germany) was mixed and incubated at room temperature in the dark for 10 min. The absorbance was measured at 540 nm using a microplate reader, and nitrite concentration was used as an indicator of NO production [41,42].

### 2.10. Liquid Chromatography-Mass Spectrometry (LC-MS) Analysis

The chemical profiling of the sample was carried out using liquid chromatography–electrospray ionization–tandem mass spectrometry (LC-ESI-MS/MS) (Agilent Technologies, Waldbronn, Germany). Separation was achieved using an ExionLC AC system coupled to a SCIEX Triple Quad 5500+ mass spectrometer equipped with an electrospray ionization (ESI) source. An Ascentis^®^ C18 column (4.6 × 150 mm, 3 µm) was used for chromatographic separation. The mobile phase consisted of solvent A (0.1% formic acid) and solvent B (acetonitrile, LC grade). The gradient elution program was set as follows: 10% B (0–1 min), 10–90% B (1–33 min), 90% B (33–37 min), and 10% B (37.1–40 min). The flow rate was maintained at 0.7 mL/min, with an injection volume of 10 µL. The LC–ESI–MS/MS system employed in the present study and metabolite annotations were based on nominal precursor ions, MS/MS fragmentation patterns, database matching (Natural Products Atlas and CFM-ID), literature comparison, and known occurrence of metabolites in Fusarium species. Compound annotation was achieved through data processing MGF files and spectral matching using MS-DIAL version 4.70, the Natural Products Atlas database, and CFM-ID version 4.0 [43,44].

### 2.11. Molecular Docking Analysis

The target protein crystal structures of human INOSOX in complex with the inhibitor AR-C95791 (PDB ID: 3E7G) which were used to evaluate the potential anti-inflammatory effect of the tentatively identified molecules were retrieved from the Protein Data Bank (https://www.rcsb.org, accessed on 23 March 2026) [45]. Their structures were prepared using Discovery Studio (v2024) by removing water molecules, adding missing hydrogen atoms, and optimizing the structure for docking. The 2D structures of the tested ligands were prepared and their energies were minimized using OpenBabel (v3.1.1) with the MMFF94 force field. The active binding sites were defined based on the coordinates of the co-crystallized ligand within the protein structure. The molecular docking algorithm was validated by redocking of the co-crystallized ligand into the active site for the reliability and reproducibility of the proposed docking algorithm [46]. The interaction between the ligands and the selected protein active sites was performed using AutoDock Vina version 1.2.0 through the PyRx 0.8 interface. The grid box was centered at coordinates X = 55.4; Y = 21.7; Z = 78.8, with dimensions of XYZ = 20 Å. The exhaustiveness parameter was set to 50 for all docking runs. The resulting binding poses and docking scores were analyzed, and then 2D and 3D interaction diagrams were generated using Discovery Studio Visualizer 2024 [47].

### 2.12. Data Analysis

All results were expressed as mean ± standard deviation (SD). IC50 and LC50 values were estimated by nonlinear dose–response regression using GraphPad Prism 10.2.3.403. Results are presented as mean ± SD of three independent experiments.

## 3. Results

### 3.1. Molecular Characterization of the Isolated Fungus

**Figure 1:**
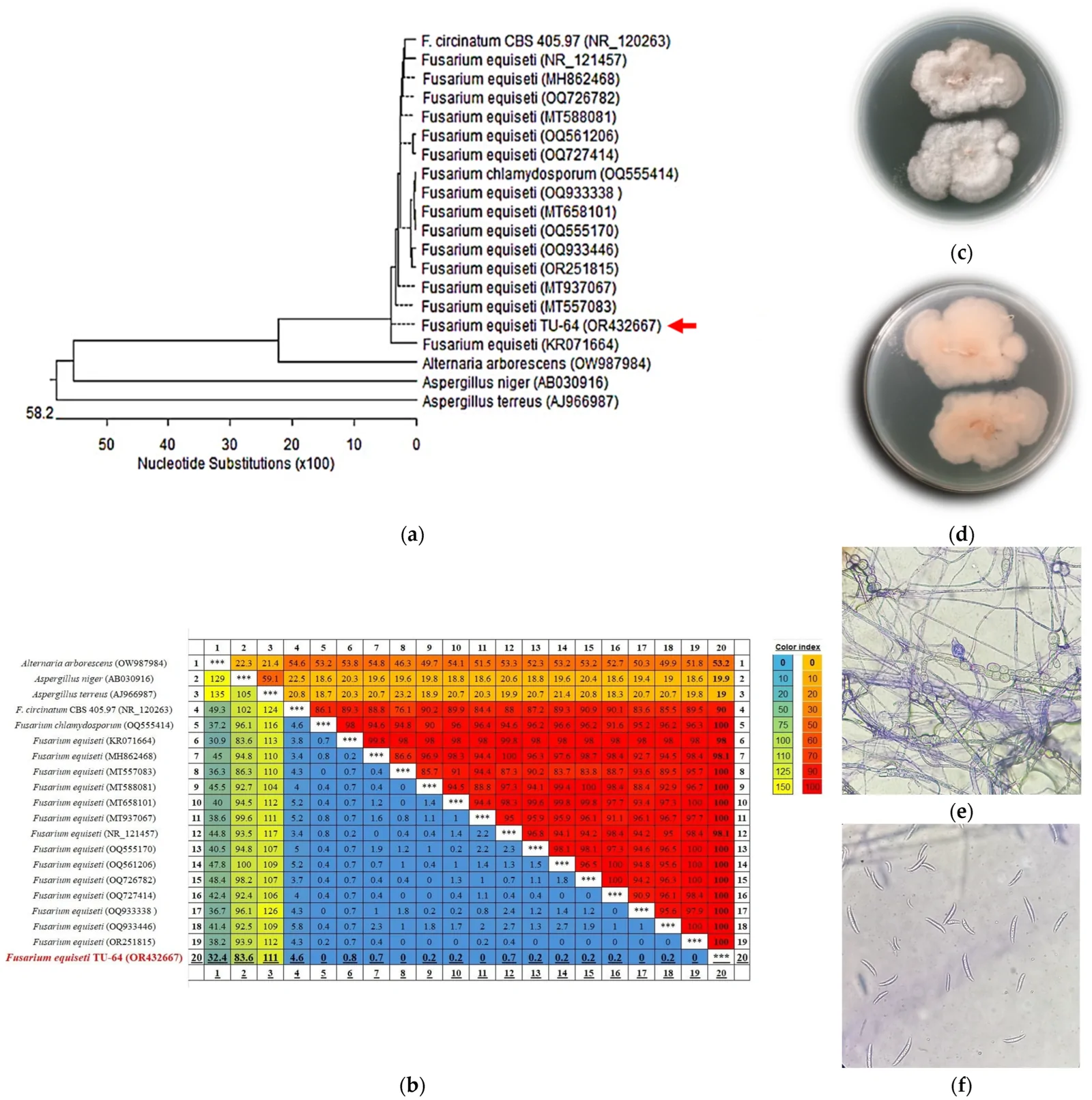
(**a**) Molecular phylogenetic tree generated from ITS region sequences of rDNA of *F. equiseti* TU-64 (arrowed) matched with closely related sequences deposited in GenBank, (**b**) heatmap (*** = 100% closest relation), (**c**) *F. equiseti* culture obverse, (**d**) reverse, (**e**) Chlamydospores, and (**f**) Macroconidia. ×400.

Molecular identification and phylogenetic analysis of the endophytic fungus *F. equiseti* were performed based on ITS region sequencing. Phylogenetic sequence analysis using closely related GenBank records indicated that the obtained ITS sequence shared 99–100% query coverage and up to 100% sequence identity with several *F. equiseti* isolates. The investigated *F. equiseti* strain showed 100% sequence similarity and 99.31% query coverage with the type of strain *F. equiseti* NRRL 26419 (NR_121457). Based on the molecular identification results, the isolated fungus was identified as *F. equiseti* and designated *F. equiseti* TU-64 with the GenBank registered accession number (OR432667). Phylogenetic analysis further confirmed the taxonomic placement of the isolate within the *F. equiseti* clade. Representative strains of *Alternaria arborescens*, *Aspergillus niger*, and *A. terreus* were included as outgroup taxa to root the phylogenetic tree (Figure 1). A heatmap analysis of the phylogenetic tree of *F. equiseti* TU-64 presenting percent similarity in the upper triangle and percent divergence in the lower triangle is illustrated in Figure 1b.

### 3.2. Total Phenolics and Alkaloids Content and Antioxidant Activity

Table 1 shows the total amounts of phenolics in the *F. equiseti* TU-64 EtOAc crude extract, exhibiting a phenolics content of 29.31 mg GAE/g, an alkaloid content of 8.43 mg AE/g, and a scavenging DPPH radical IC_50_ value of 2.18 mg/mL. The results of BHT, as a standard positive control, demonstrated an antioxidant scavenging activity of 0.15 mg/mL IC_50_ value.

### 3.3. Impact of F. equiseti Extract on Acetylcholine and Butyrylcholinesterases

**Figure 2:**
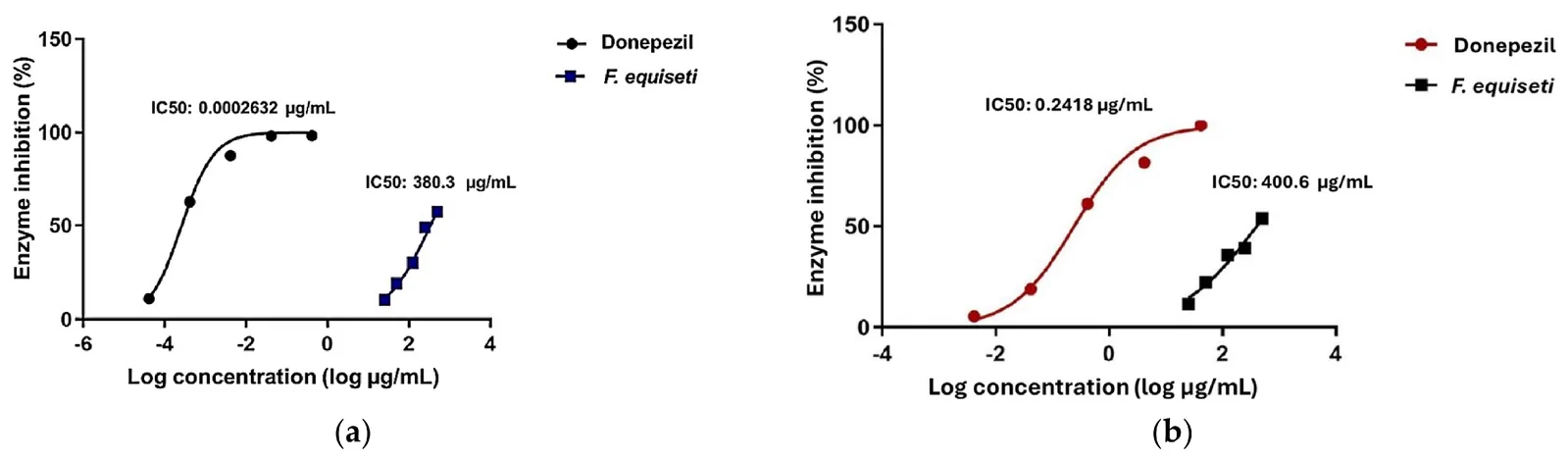
Inhibitory activity of the EtOAc extract of *F. equiseti* TU-64 against (**a**) acetylcholinesterase (AChE) and (**b**) butyrylcholinesterase (BChE).

The EtOAc extract of *F. equiseti* TU-64 exhibited an IC_50_ value of 380.30 ± 22.87 μg/mL against AChE (Figure 2a, Table S1) and 400.60 ± 9.77 μg/mL against BChE (Figure 2b, Table S1). Compared with the reference inhibitor donepezil, the fungal extract demonstrated very weak cholinesterase inhibitory activity.

### 3.4. Cytotoxicity and Brine Shrimp Lethality Assay

**Figure 3:**
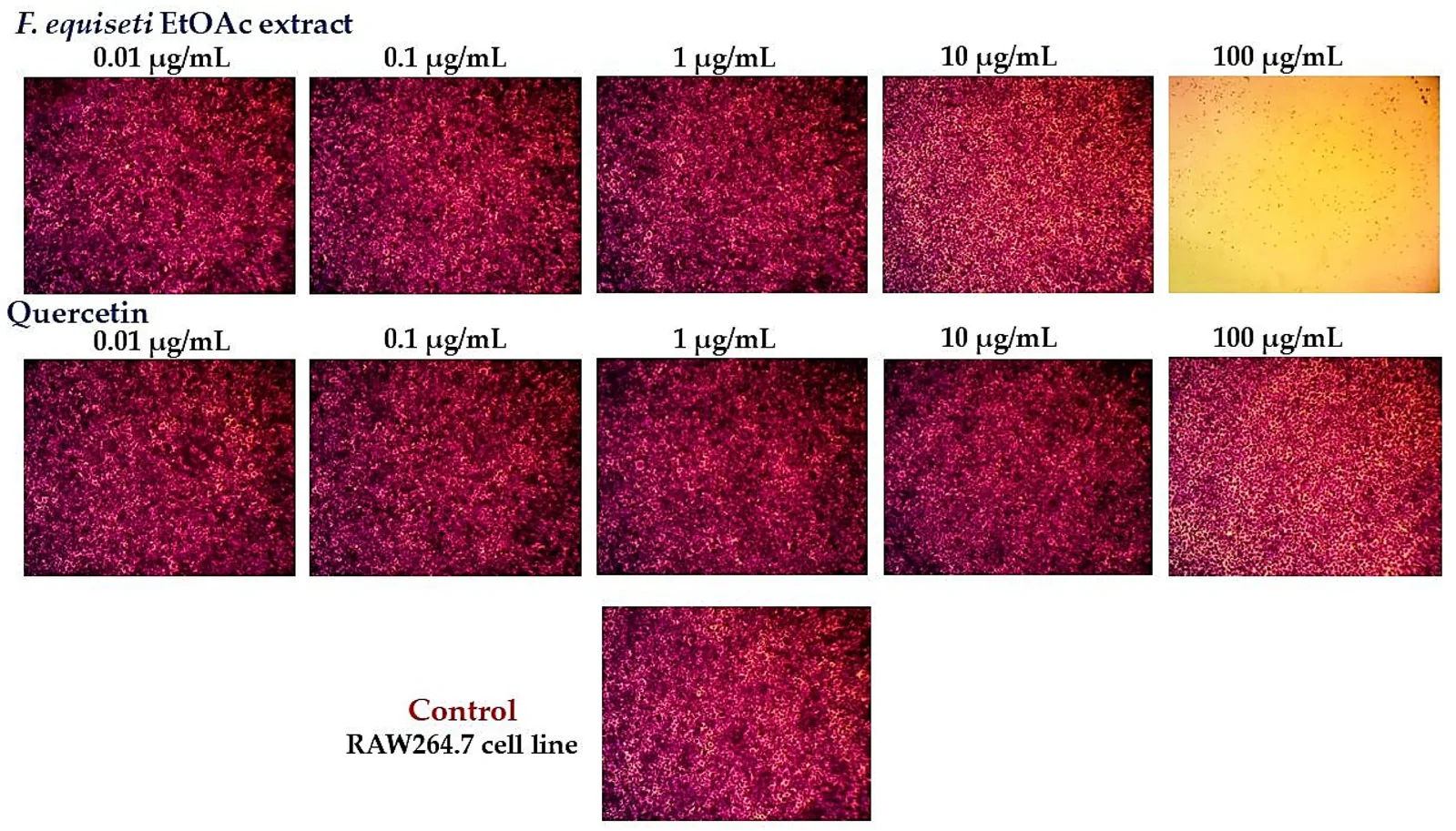
Representative optical micrographs showing the cytotoxic effects of the EtOAc extract of *F. equiseti* TU-64 and quercetin on RAW264.7 cells. Images were captured using an optical microscope at ×100 magnification.

The cytotoxic activity of the F. equiseti TU-64 EtOAc extract was evaluated against the RAW264.7 murine macrophage cell line. The IC_50_ values were determined from the corresponding dose–response curves. The fungal extract exhibited an IC_50_ value of 49.95 μg/mL, whereas the positive control, quercetin, showed an IC_50_ value of 529.21 μg/mL (Table 2 and Figure 3). The cytotoxic potential of the extract was further assessed using the A. salina brine shrimp lethality assay. The extract exhibited an LC_50_ value of 1.86 mg/mL, indicating relatively low toxicity toward brine shrimp larvae (Table 2).

### 3.5. In Vitro Anti-Inflammatory Assay

**Figure 4:**
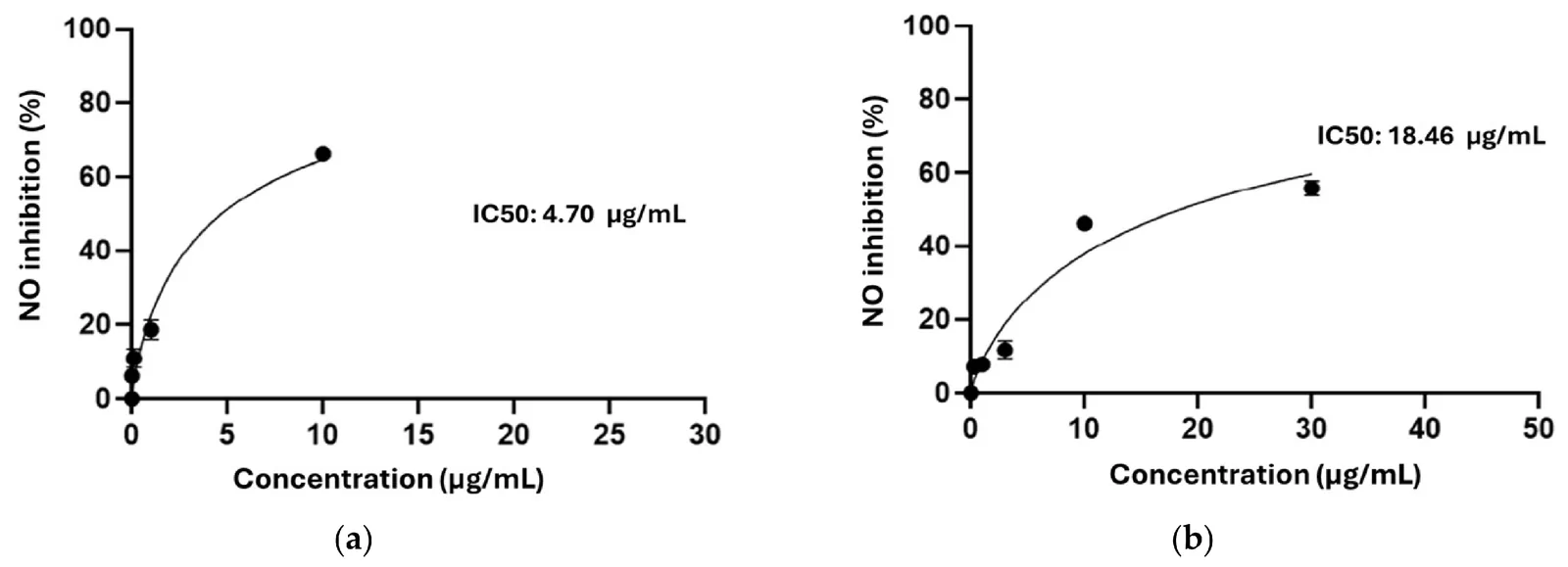
Anti-inflammatory activity (inhibition of NO production) of (**a**) *F. equiseti* TU-64 EtOAc extract and (**b**) quercetin.

The anti-inflammatory activity of the EtOAc extract of *F. equiseti* TU-64 was evaluated by measuring its inhibitory effect on nitric oxide (NO) production in lipopolysaccharide (LPS)-stimulated RAW264.7 macrophages. As shown in Figure 4, treatment with the fungal extract significantly suppressed NO production in LPS-induced RAW264.7 cells in a concentration-dependent manner. The *F. equiseti* extract exhibited an IC_50_ value of 4.70 ± 0.86 μg/mL for NO inhibition, indicating potent anti-inflammatory activity. In comparison, the reference anti-inflammatory compound quercetin showed an IC_50_ value of 18.46 ± 1.66 μg/mL. These results suggest that the EtOAc extract of *F. equiseti* TU-64 possesses strong anti-inflammatory activity and was more effective than quercetin in inhibiting NO production under the experimental conditions employed.

### 3.6. LC-MS/MS Analysis

**Figure 5:**
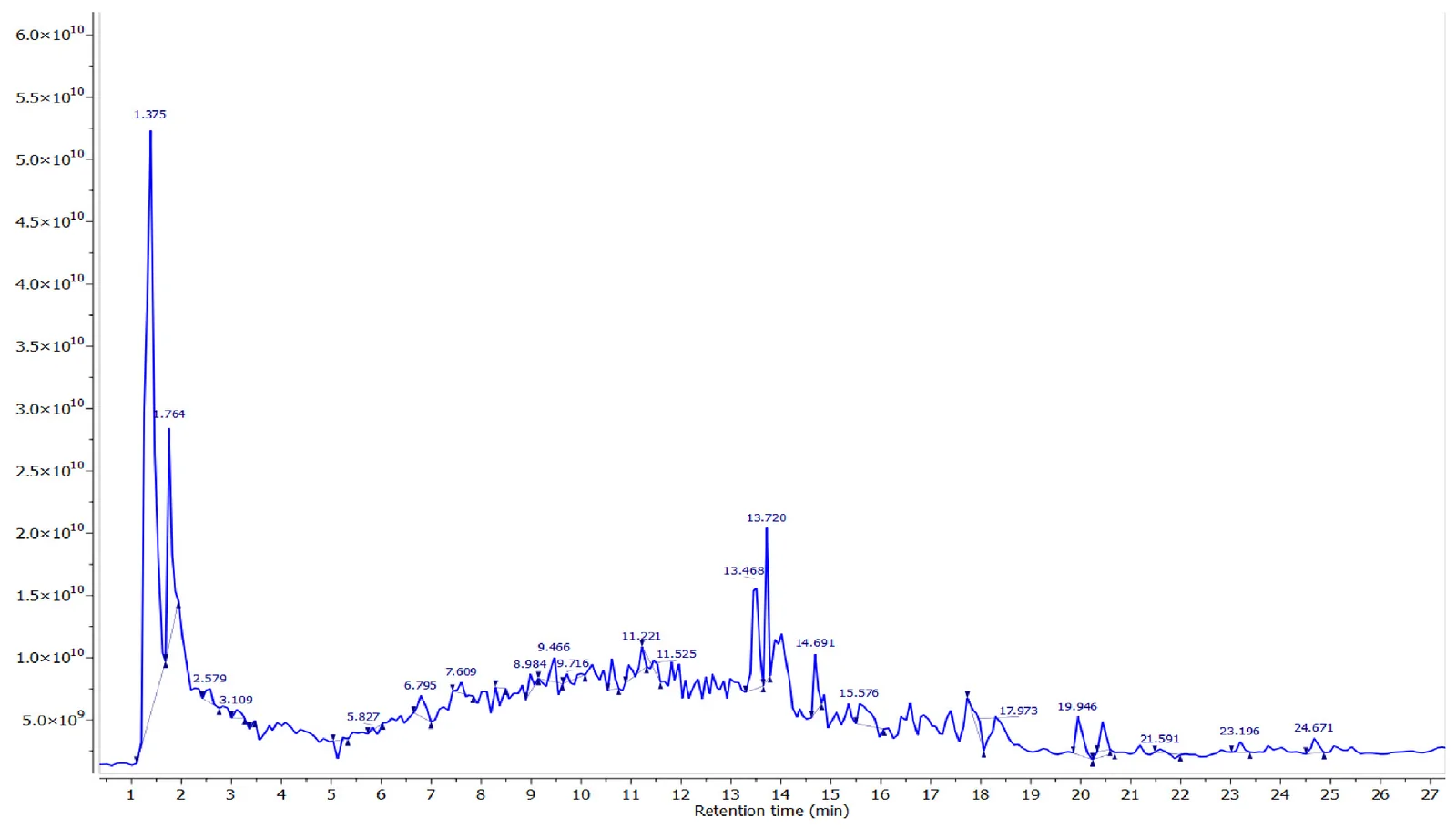
TIC chromatogram (LC–MS/MS ESI) of the ethyl acetate extract of *F. equiseti* positive mode.

**Figure 6:**
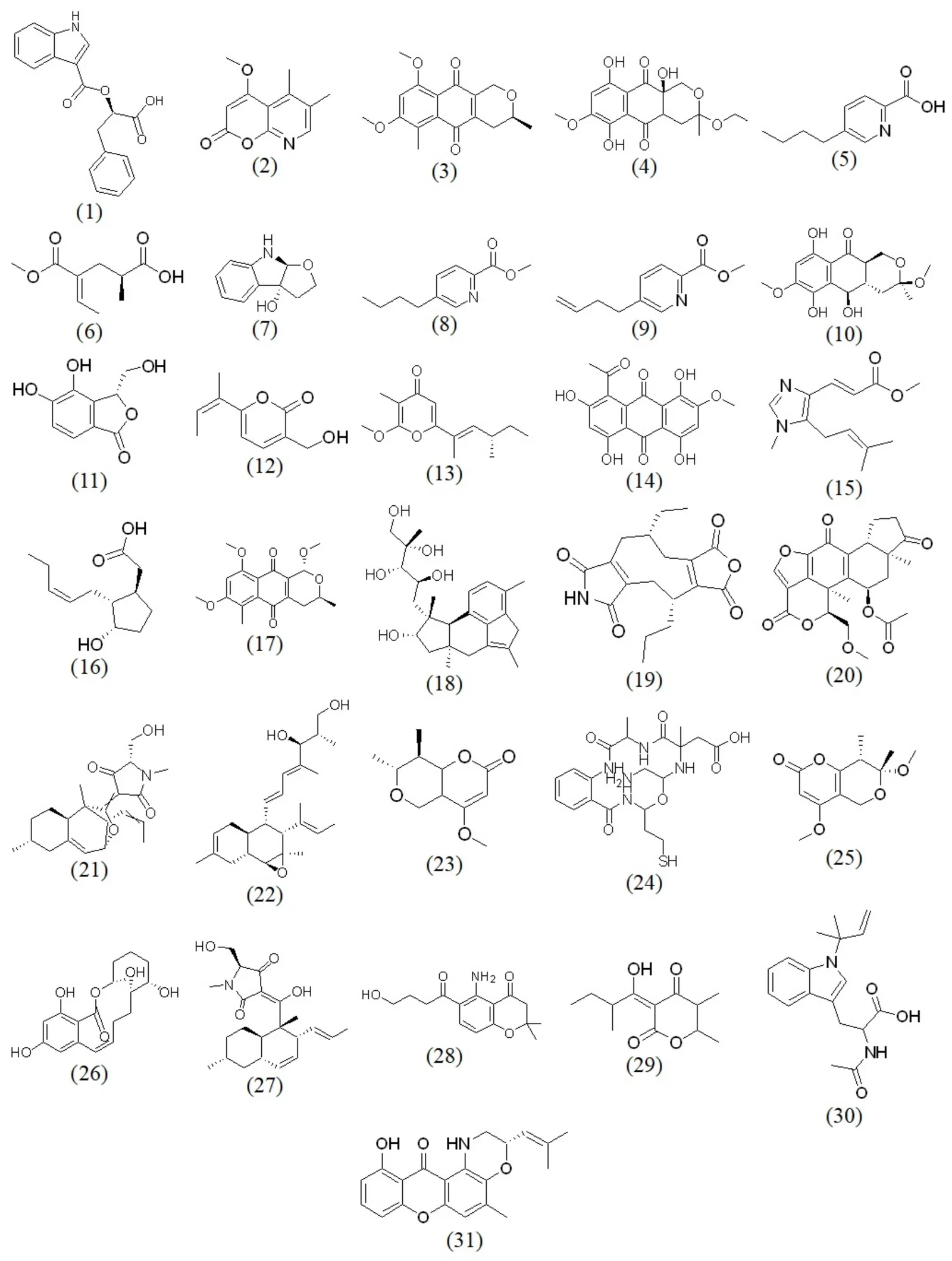
Structures of metabolites (1–31) putatively identified via LC-MS/MS analysis of the *F. equiseti* EtOAc extract.

UHPLC–ESI–MS analysis of the fungal extract revealed a broad range of secondary metabolites. The tentative identification of observed peaks revealed the detection of 31 compounds (Table 3 and Figure 5 and Figure 6). The results showed the presence of an ion peak at *m*/*z* 310.07 [M + H]^+^ consistent with the chemical formula (C_18_H_15_NO_4_) with a retention time (RT) of 2.05 min. The ion fragment peak at *m*/*z* 264 corresponds to CO_2_ loss (−44 Da), consistent with the even-electron rule. Subsequent fragments at *m*/*z* 162 and 144 arise from α-cleavage adjacent to the indole nitrogen. The mass fragment ions (*m*/*z* 121, 105, 103) represent stabilized benzyl/phenyl cations. The compound was tentatively identified as fusariumindole C. The positive ion peak at *m*/*z* 206.08 [M + H]^+^ correlating to the chemical formula (C_11_H_11_NO_3_) with an RT of 2.90 min. The ion fragment peaks at *m*/*z* 190 and 188 correspond to sequential losses of CH_4_ and H_2_O. The ion fragment at *m*/*z* 178 arises from CO elimination, while the ion at *m*/*z* 125 represents a stabilized heteroaromatic fragment. The compound was tentatively identified as acuminatopyrone.

Astropaquinone D (C_17_H_18_O_5_; *m*/*z* 303.10, 3.10 min) fragments via sequential H_2_O and CO losses (*m*/*z* 285, 271, 257, 243), the ions at *m*/*z* 215 and 179 arise from retro Diels–Alder cleavage, producing conjugated fragments stabilized by resonance. O-ethylhydroxydihydrofusarubin (C_17_H_20_O_8_; *m*/*z* 353.12, 3.62 min) showed *m*/*z* 335 (−H_2_O) and 307 (−C_2_H_6_O), both neutral losses. Fragments at *m*/*z* 293, 289, 253, and 249 reflect sequential CO eliminations and α-cleavage near carbonyl groups. The results showed the presence of a positive ion peak at *m*/*z* 180.08 [M + H]^+^ in agreement with the chemical formula (C_10_H_13_NO_2_) with an RT of 3.82 min. Fragment ions at *m*/*z* 162 and 138 correspond to losses of H_2_O and CO_2_, respectively, followed by *m*/*z* 120 from further ring cleavage. Charge retention on the pyridine ring and the odd mass confirm one nitrogen atom. The compound was tentatively identified as fusaric acid, and the results showed a positive ion peak at *m*/*z* 187.02 [M + H]^+^ in accordance with the chemical formula (C_9_H_14_O_4_) with an RT of 5.20 min. Fragment ions at *m*/*z* 155 and 141 indicate losses of CH_3_OH and CO_2_. Additional fragments (*m*/*z* 137, 127, 123, 111, 109) arise from β-cleavage and dehydration. The compound was tentatively identified as fusarin H.

The compound (-)-3α-hydroxyfuroindoline was tentatively identified according to the presence of a positive ion peak at *m*/*z* 178.07 [M + H]^+^ matching the chemical formula (C_10_H_11_NO_2_) with an RT of 6.61 min. Fragment ions at *m*/*z* 160 and 150 correspond to losses of H_2_O and CO, followed by *m*/*z* 142 and 134 via α-cleavage adjacent to nitrogen, which forms stabilized iminium ions. Fusaric acid methyl ester (C_11_H_15_NO_2_; *m*/*z* 194.10, 7.03 min) fragments correspond to *m*/*z* 162 (−CH_3_OH) and 132 (−CO_2_), consistent with ester cleavage and even-electron neutral loss. The ion fragment at *m*/*z* 120 arises from pyridine ring stabilization. 9,10-dehydrofusaric acid methyl ester (C_11_H_13_NO_2_; *m*/*z* 192.08, at 7.56 min) exhibits fragment ions at 174 (−H_2_O) and 160 (−CO), followed by *m*/*z* 150, 132, 118, and 105 through allylic cleavage enhanced by unsaturation, producing resonance-stabilized ions. 5-hydroxy-3-methoxydihydrofusarubin A (C_16_H_20_O_7_; *m*/*z* 325.14, 7.79 min) fragments via sequential losses of CH_3_OH and CO (*m*/*z* 293, 275), followed by several fragmentation (*m*/*z* 265, 261, 251, 239, 211, 197) via α-cleavage and inductive oxygen effects, yield stabilized aromatic ions.

The results showed the presence of a positive ion peak at *m*/*z* 197.06 [M + H]^+^ in accordance with the chemical formula (C_9_H_8_O_5_) with an RT of 8.11 min. Fragment ions at *m*/*z* 179 and 169 correspond to losses of H_2_O and CO, with further fragments arising from ring cleavage and decarboxylation. The compound was tentatively identified as fusagunolic A. Fusarpyrone A was tentatively identified upon the presence of a positive ion peak at *m*/*z* 181.06 [M + H]^+^ corresponding to the chemical formula (C_10_H_12_O_3_) with an RT of 8.33 min. Fragment ions at *m*/*z* 161 and 149 correspond to losses of H_2_O and CO, followed by multiple fragments due to pyrone ring cleavage. The presence of an ion peak at *m*/*z* 237.10 [M + H]^+^ corresponding to the chemical formula (C_14_H_20_O_3_) was utilized to identify fusarester A tentatively with an RT of 8.54 min. Fragment ions at *m*/*z* 219 and 205 correspond to losses of H_2_O and CO, with additional fragments resulting from ester α-cleavage and chain fragmentation.

Karimunone A was tentatively identified according to the presence of an ion peak at *m*/*z* 345.03 [M + H]^+^ corresponding to the chemical formula (C_17_H_12_O_8_) with an RT of 8.80 min. Fragment ions at *m*/*z* 327, 309, and 299 correspond to sequential CO losses, followed by retro Diels–Alder fragmentation products. The results showed the presence of an ion peak at *m*/*z* 235.14 [M + H]^+^ consistent with the chemical formula (C_13_H_18_N_2_O_2_) with an RT of 9.13 min. Fragment ions at *m*/*z* 217 and 193 correspond to losses of H_2_O and NH_3_, with further ions reflecting iminium stabilization. The compound was tentatively identified as fungerin. The presence of an ion peak at *m*/*z* 213.14 [M + H]^+^, consistent with the chemical formula (C_12_H_20_O_3_) with an RT of 9.71 min and fragment ions at *m*/*z* 195 and 177, and corresponding to losses of H_2_O and CO followed by chain fragmentation via β-cleavage, tentatively identified the compound as 7-iso-cucurbic acid. Tentatively identified 6-hydroxy-astropaquinone B displayed the presence of an ion peak at *m*/*z* 333.12 [M + H]^+^ in accordance with the chemical formula (C_18_H_20_O_6_) with an RT of 9.84 min. Meanwhile, fragment ions arise from sequential losses typical of quinone systems.

Neomangicol C (C_25_H_36_O_5_; *m*/*z* 417.10, 10.60 min) demonstrated fragments at *m*/*z* 399 and 381 (−H_2_O losses), followed by *m*/*z* 371, 293, 267, 251, and 211 through terpenoid chain cleavage and rearrangements. (-)-byssochlamic acid imide (C_18_H_21_NO_5_; *m*/*z* 332.10, 11.11 min) yields fragments ion at *m*/*z* 314 (−H_2_O) and 304 (−CO), with *m*/*z* 285 and 213 arising from imide-directed α-cleavage, consistent with nitrogen stabilization. Wortmannin (C_23_H_24_O_8_; *m*/*z* 429.10, 11.90 min) shows sequential losses (*m*/*z* 411, 397, 369, 351, 349, 341), followed by *m*/*z* 291, 259, and 186 via extensive rearrangements and ring fragmentation, including RDA. Decalintetracid A (C_22_H_29_NO_4_; *m*/*z* 372.12, 14.43 min) fragments to *m*/*z* 354 (−H_2_O), 344 (−CO), and further to *m*/*z* 298, 264, 229, 203, 189 via α-cleavage and chain fragmentation. Fusarielin H (C_25_H_38_O_3_; *m*/*z* 387.21, 16.09 min) produces ion peaks at *m*/*z* 309, 263, 241, 233, 201, 187, 175, 153, and 135 due to extensive C–C cleavage and allylic fragmentation, typical of polyketides. Fusaritricin A/B (C_11_H_16_O_4_; *m*/*z* 213.10, 16.20 min) shows fragment peaks at *m*/*z* 197 (−H_2_O), 185 (−CO), and further fragments (*m*/*z* 179, 169, 155, 141) via sequential neutral losses and α-cleavage. Fusarithioamide B (C_20_H_29_N_5_O_6_S; *m*/*z* 468.18, 16.23 min) yields *m*/*z* 450 (−H_2_O), 391, and 344 via cleavage adjacent to sulfur and nitrogen, producing stabilized heteroatom-centered ions. The results presented the presence of an ion peak at *m*/*z* 241.07 [M + H]^+^ matching with the chemical formula (C_12_H_16_O_5_) with an RT of 16.46 min. Fragment ions arise from lactone ring opening and α-cleavage. The compound was tentatively identified as fusarilactone A.

The identified compound 5′-hydroxyzearalenol showed the presence of an ion peak at *m*/*z* 337.17 [M + H]^+^ consistent with the chemical formula (C_18_H_24_O_6_) with an RT of 16.70 min. Fragment ions result from macrocyclic cleavage and retro Diels–Alder reactions. The data introduced the presence of an ion peak at *m*/*z* 374.27 [M + H]^+^ corresponding to the chemical formula (C_22_H_31_NO_4_) with an RT of 17.53 min. Fragment ions reflect nitrogen-directed cleavage and polyketide fragmentation. This compound was tentatively identified as equisetin. The results revealed the presence of an ion peak at *m*/*z* 278.10 [M + H]^+^ corresponding to the chemical formula (C_15_H_19_NO_4_) with an RT of 19.24 min. Fragment ions arise from α-cleavage near amino and carbonyl groups, forming iminium ions. The compound was tentatively identified as 2,2-dimethyl-5-amino-6-(4′-hydroxylbutyryl)-4-chromone. Fujikurin D (C_12_H_18_O_4_; *m*/*z* 227.13, 21.25 min) fragments to *m*/*z* 171, 143, 129, 123, and 109 via chain cleavage and dehydration. Ac-r-N-DMAT (C_18_H_22_N_2_O_3_; *m*/*z* 315.17, 24.71 min) shows *m*/*z* 297 (−H_2_O), 255, 227, and 201 via nitrogen-directed fragmentation and indole stabilization, consistent with the nitrogen rule. Fusarioxazin (C_20_H_19_NO_4_; *m*/*z* 338.14, 25.48 min) fragments correspond to *m*/*z* 310, 296, 282, 264, 256, 240, and 225 fragments via sequential neutral losses and aromatic cleavage. Metabolite annotations were generated by processing the acquired MS/MS data with MS-DIAL and comparing the spectra and candidate structures with the Natural Products Atlas, CFM-ID predictions, and published fragmentation data. Candidate assignments were manually evaluated using precursor-ion information, molecular formula, product-ion patterns, characteristic neutral losses, chemical plausibility, and previously reported fungal occurrence. Because authentic standards were not analyzed under identical chromatographic and mass-spectrometric conditions, the reported compounds represent putative annotations rather than unequivocal identifications. The annotations were therefore classified primarily as MSI Level 2, depending on the available spectral evidence and further purification and spectroscopic characterization, particularly by NMR, will be required to confirm the proposed structures.

### 3.7. Molecular Docking

**Figure 7:**
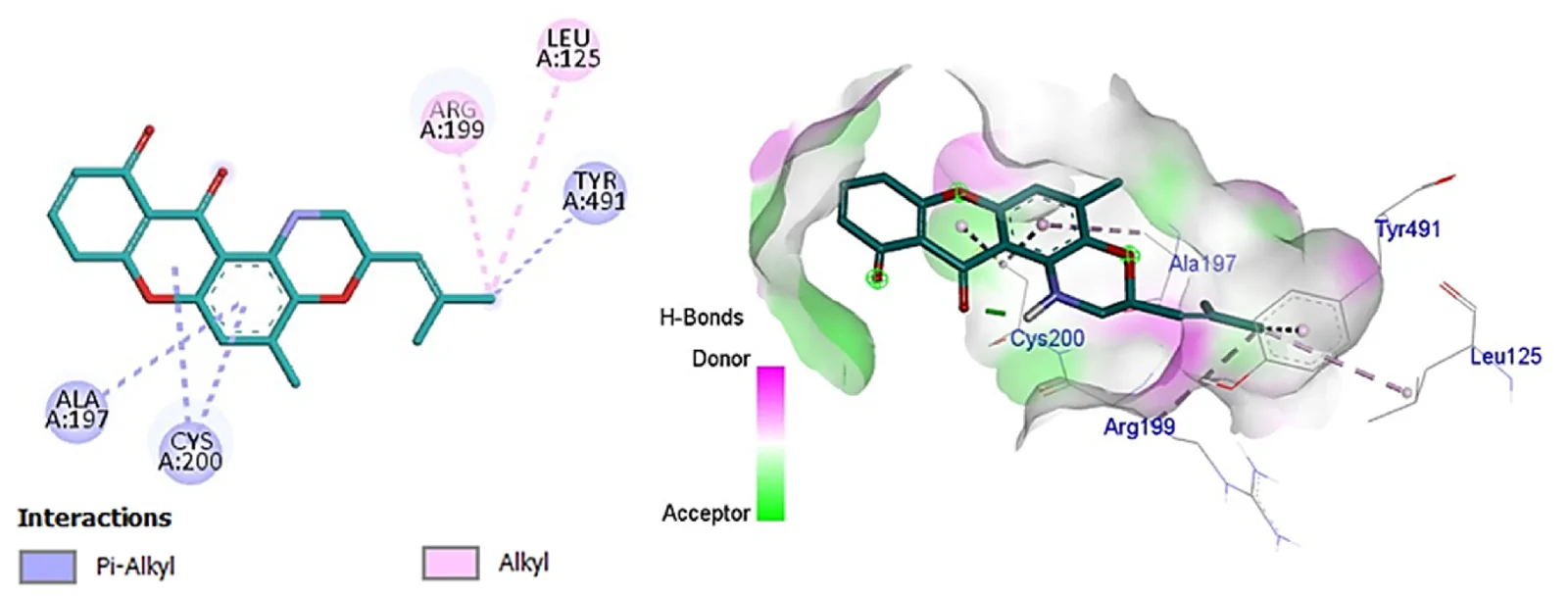
Two-dimensional (**Left**) and three-dimensional (**Right**) poses and interactions of fusarioxazin in 3E7G active site.

**Figure 8:**
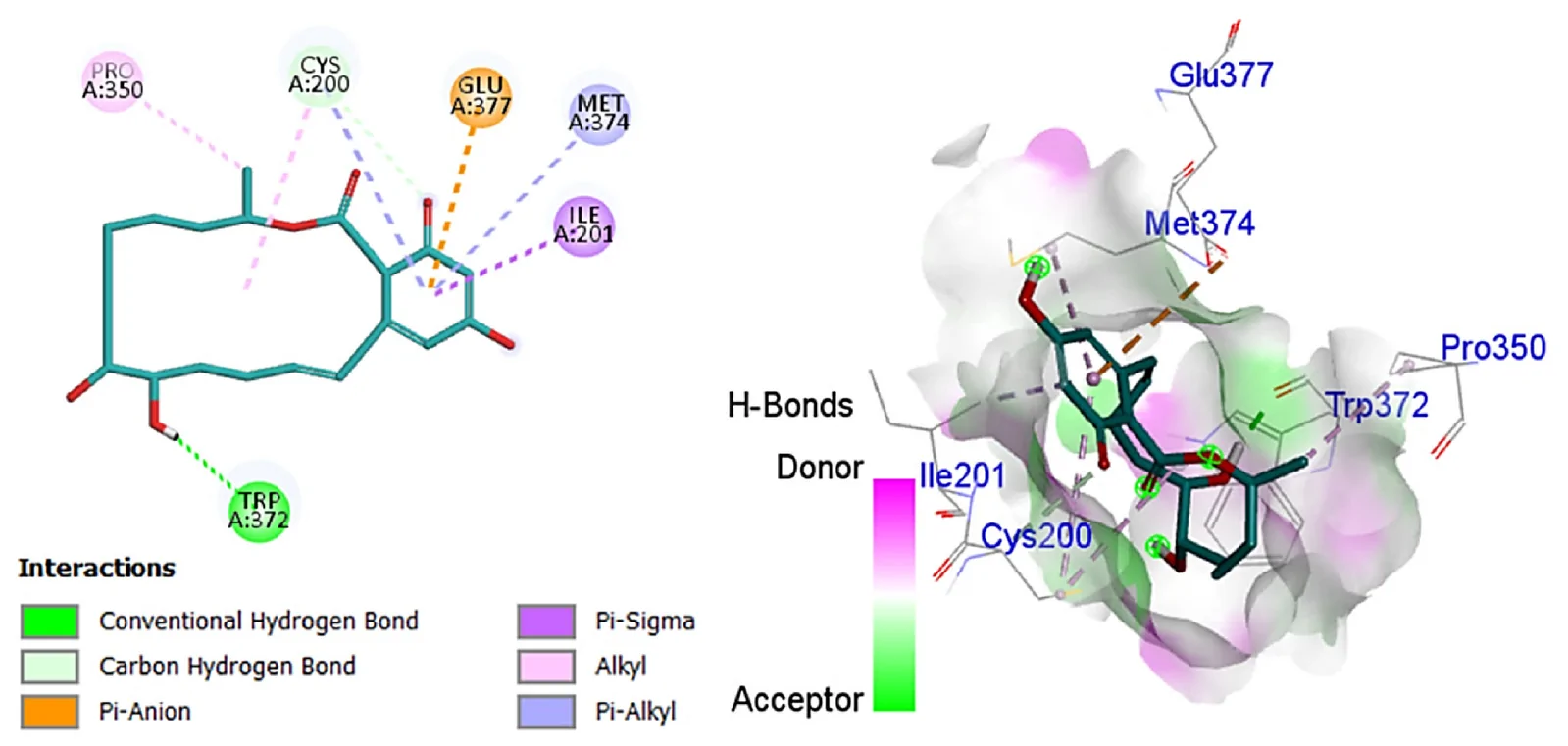
Two-dimensional (**Left**) and three-dimensional (**Right**) poses and interactions of 5′-hydroxyzearalenol in 3E7G active site.

**Figure 9:**
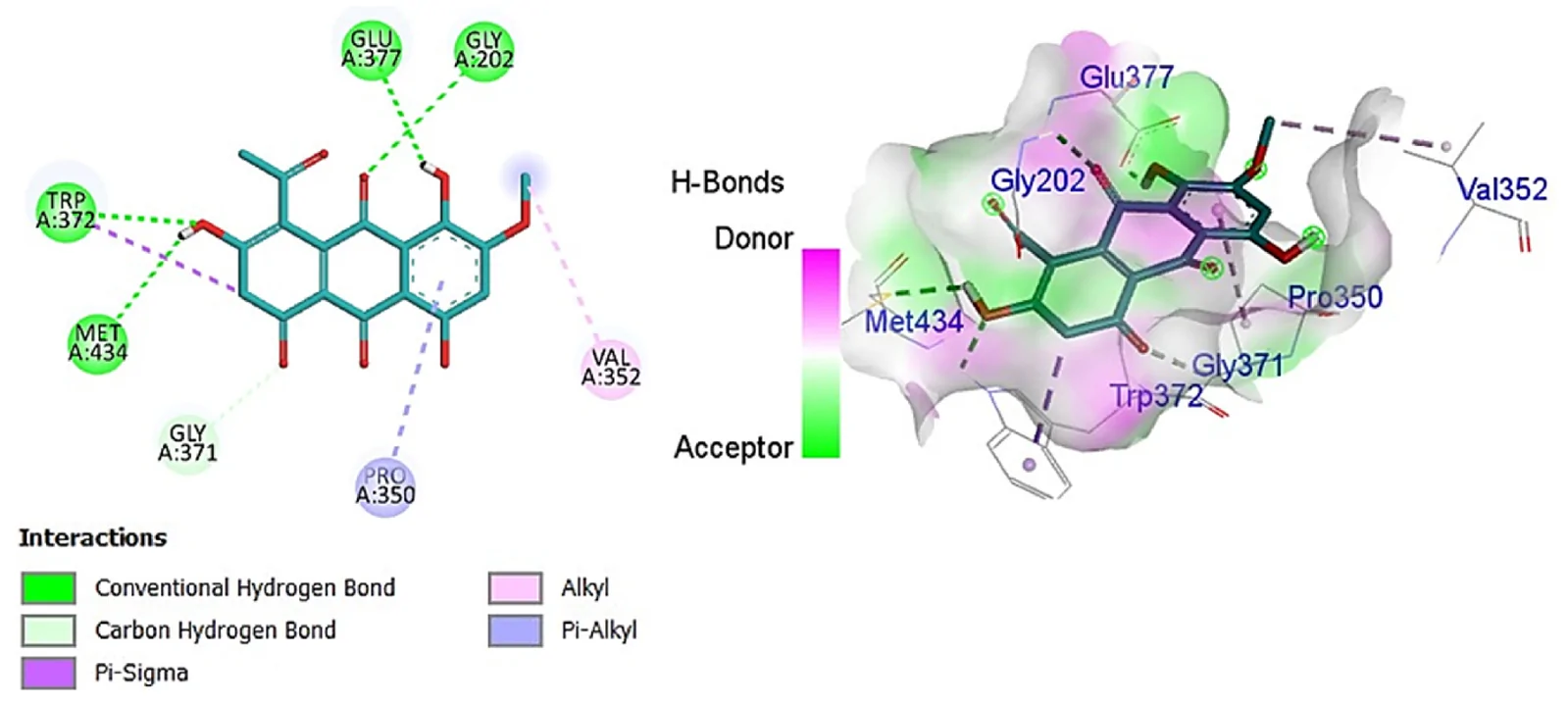
Two-dimensional (**Left**) and three-dimensional (**Right**) poses and interactions of karimunone A in 3E7G active site.

The molecular docking study was performed to assess the binding affinities and interaction profiles of a group of compounds with inhibition activity of Nitric Oxide Synthase (iNOS), represented by its crystal structure (PDB ID: 3E7G) using candidate structures derived from tentative LC–MS/MS annotations. iNOS is a crucial enzyme which has a definite role in inflammatory processes, and its suppression is a significant therapeutic target. The docking results revealed considerable variation in binding affinities among the tested compounds, indicating differences in their potential inhibitory activities toward iNOS. The calculated binding energies for all investigated ligands are presented in Table 4. The co-crystallized reference ligand, AR-C95791, exhibited a binding affinity of −6.7 kcal/mol (Table 4). Several compounds remarkably exhibited superior binding affinities than the reference, suggesting their potential as iNOS inhibitors. The results putatively identified several compounds with promising binding potential with inhibitory site against iNOS (PDB ID: 3E7G). Fusarioxazin came first as the most favorable binder, followed closely by 5′-hydroxyzearalenol and karimunone A at the same level. Consequently, the top-performing ligands were analyzed to elucidate their specific interaction mechanisms within the iNOS active site. Figure 7, Figure 8 and Figure 9 illustrate the 2D and 3D interaction profiles for Fusarioxazin, 5′-Hydroxyzearalenol, and Karimunone A, respectively.

Fusarioxazin exhibited the highest binding affinity score among all tested compounds with a docking score of −8.8 kcal/mol. Its interaction profile within the iNOS active site (PDB ID: 3E7G), illustrated in Figure 7, revealed a complex network of stabilizing interactions. Key amino acid residues involved in ligand binding included Arg199, Leu125, Ala197, Cys200, and Tyr491, which predominantly interacted with fusarioxazin through alkyl and π–alkyl interactions. These hydrophobic contacts contribute significantly to the stabilization of the ligand within the binding pocket. Collectively, the observed interactions indicate a favorable binding mode, suggesting a high degree of complementarity between fusarioxazin and the iNOS active site, which may account for its superior binding affinity compared with the other tested compounds.

5′-hydroxyzearalenol exhibited a strong binding affinity against iNOS, with a docking score of −8.5 kcal/mol. As shown in Figure 8, its binding mode was characterized by a diverse array of interactions within the enzyme active site. A conventional hydrogen bond was formed with Trp372, while a carbon–hydrogen bond was observed with Cys200. In addition, π–anion interactions with Glu377, a π–sigma interaction with Ile201, and alkyl interactions with Pro350 contributed to ligand stabilization. Further stabilization was provided by π–alkyl interactions involving Cys200 and Met374. The combination of hydrogen-bonding and hydrophobic interactions indicates a favorable binding orientation and strong molecular recognition of 5′-hydroxyzearalenol within the iNOS active site, supporting its potential as a promising iNOS inhibitor.

Both 5′-hydroxyzearalenol and karimunone A had the same binding affinity score of −8.5 kcal/mol, indicating a strong interaction with the iNOS active site, with an interaction profile (Figure 9) that includes multiple conventional hydrogen bonds with Glu377, Gly202, Trp372, and Met434. As illustrated in Figure 9, its binding mode was characterized by an extensive network of interactions, including several conventional hydrogen bonds with Glu377, Gly202, Trp372, and Met434, which likely play a major role in stabilizing the ligand–protein complex. In addition, a carbon–hydrogen bond was observed with Gly371. Hydrophobic interactions further contributed to binding stability, including a π–sigma interaction with Trp372, an alkyl interaction with Val352, and a π–alkyl interaction with Pro350. The combination of multiple hydrogen bonds and hydrophobic contacts suggests a stable and highly specific binding mode of karimunone A within the iNOS active site, supporting its potential as a promising iNOS inhibitory compound.

Because the differences among their calculated scores were small, these compounds should be considered similarly ranked computational candidates rather than assigned a definitive order of affinity or potency. These findings provide a structural hypothesis that may support prioritization for subsequent isolation and experimental assessment.

These findings proved valuable insights into the structural necessities for effective iNOS inhibition and can serve as a guiding framework for rational design of novel therapeutic agents for inflammatory situations.

The mechanistic relationship between the observed reduction in nitric oxide production and direct iNOS inhibition was not experimentally established. Reduced nitrite levels in LPS-stimulated RAW264.7 cells may result from several mechanisms, including altered iNOS expression, effects on upstream inflammatory signaling, direct enzymatic inhibition, or nonspecific cytotoxicity. Therefore, direct iNOS enzyme assays, protein-expression analysis, and testing of purified, structurally confirmed compounds are required to validate the proposed mechanism. Although LC–MS/MS dereplication enabled rapid annotation of metabolites potentially responsible for the observed biological activities, these annotations remain tentative. Therefore, the molecular docking results should likewise be interpreted as preliminary computational evidence supporting potential ligand–target interactions rather than proof of biological mechanism. Future studies will focus on bioactivity-guided isolation of the major metabolites followed by complete structural elucidation and biological validation.

## 4. Discussion

Endophytic fungi are an important and assorted group of microorganisms that establish symbiotic relationships with their host plants and play important ecological and biological roles. They are well known for producing secondary metabolites with great therapeutic potential. Several novel antibiotics, antifungal agents, immunosuppressants, anti-inflammatory and anticancer compounds have been identified and developed as promising therapeutic candidates [77].

In the present study, *F. equiseti* was isolated as an endophytic fungus from the bulbs of *P. maritimum*. Identification of *F. equiseti* TU-64 was confirmed using ITS gene sequencing [77]; a multilocus molecular phylogenetic approach is therefore necessary for accurate identification of *Fusarium* species [78,79]. There are few publications on *P. maritimum* endophytic fungi. Elbermawi et al. [80] isolated *Alternaria* sp. from fresh *P. maritimum* leaves and found that it has antidiabetic properties. El-Metwally et al. [81] isolated *Mucor hiemalis* and *Aspergillus niger* from *P. maritimum* bulbs, and *Alternaria alternata*, *A. pluriseptata*, *Cladosporium cladosporioides*, *C. nigrellam*, *Penicillium marneffei*, and *Pythium nayoroerse* from the leaves, with *A. pluriseptata* exhibiting antimicrobial capabilities. Hassane et al. [82] and Khalaf et al. [83] isolated *P. chrysogenum* from *P. maritimum* seeds, *Aspergillus candidus* from the stem [84], and *A. versicolor* [85] and *P. chrysogenum* [86] from the leaves.

In the present study, the isolated *F. equiseti* TU-64 EtOAc showed total phenolic content of 29.31 mg GAE/g extract and a total alkaloid yield of 8.43 mg AE/g extract, and a scavenging DPPH radical IC_50_ value of 2.18 mg/mL. This agrees with reports that phenolic-rich extracts of *Fusarium* spp. exhibit notable antioxidant activity [87,88]. Similarly, antioxidant activity in the host plant *P. maritimum* has been associated with its phenolic and flavonoid constituents [4]. These findings support a possible contribution of phenolic metabolites to the antioxidant activity of *F. equiseti* TU-64.

The principal finding of this study is the marked suppression of NO production by the EtOAc extract of the endophytic *F. equiseti* TU-64 in LPS-stimulated RAW264.7 macrophages. The extract produced an NO-inhibition IC_50_ of 4.70 ± 0.86 μg/mL, compared with 18.46 ± 1.66 μg/mL for quercetin under the same experimental conditions. This result is substantially stronger than the extract’s DPPH radical-scavenging activity and its cholinesterase inhibition and therefore provides the clearest biological signal in the screening dataset. Importantly, the NO-inhibition concentration is below the extract’s RAW264.7 cytotoxicity IC_50_ (49.95 μg/mL), which supports a concentration window in which suppression of NO cannot be explained solely by generalized loss of cell viability. Nevertheless, dedicated viability measurements at each concentration used in the NO assay would strengthen this interpretation.

UHPLC–ESI–MS analysis of the fungal extract revealed a broad range of secondary metabolites. The tentative identification of observed peaks revealed the detection of 31 compounds. Several annotated metabolites have previously been associated with anti-inflammatory or related biological effects. For example, fusarin H has been reported to suppress NO production in LPS-activated RAW264.7 cells [53], whereas fusagunolic A showed only low inhibition of NO production in the same cell model [57].

Molecular docking is a widely used technique which aims to predict the optimal pathway by which one molecule can attach to a target, ultimately leading to the development of a stable complex. The assessment of binding efficacy to the appropriate target binding site plays a key role in pharmaceutical development and in comprehending the fundamental biochemical mechanisms [89].

The anti-inflammatory focus is mechanistically consistent with the computational analysis. iNOS is induced in activated macrophages and catalyzes sustained NO production during inflammatory responses [90,91]. Regulation of NO synthesis via iNOS occurs mainly at the transcriptional and translational levels, and once activated, the enzyme continuously produces high levels of NO until the substrate is depleted. NO is further involved in modulating the expression and activity of oncogenes that play essential roles in cell cycle regulation and apoptosis [92].

For this reason, iNOS was selected as the docking target. Among the 31 tentatively annotated metabolites, fusarioxazin had the most favorable docking score (−8.8 kcal/mol), followed by 5′-hydroxyzearalenol and karimunone A (−8.5 kcal/mol). These docking results do not demonstrate iNOS inhibition, but they provide testable hypotheses for prioritizing compounds during bioactivity-guided fractionation as the measured biological effect belongs to the crude extract.

The complementary screening assays help define the specificity and limitations of the extract. Cholinesterase inhibition was included initially because *P. maritimum* and the Amaryllidaceae are well known for alkaloids such as galanthamine and because endophytic fungi may provide chemically diverse metabolites associated with their host environment [5,93,94,95,96]. However, the *F. equiseti* TU-64 extract showed only very weak inhibition of AChE and BChE (IC_50_ = 380.30 ± 22.87 and 400.60 ± 9.77 μg/mL, respectively). These negative/weak findings do not support cholinesterase inhibition as a major pharmacological property of this extract; accordingly, the data are reported as supplementary screening results.

The present findings indicate that the ethyl acetate extract of the investigated endophytic *F. equiseti* TU-64 strain exhibited measurable antioxidant, and nitric oxide-inhibitory activities under the applied experimental conditions. However, further studies involving bioassay-guided fractionation and confirmation of the active constituents will be conducted to validate the proposed structures and confirm the predicted molecular interactions.

## 5. Conclusions

This study identifies suppression of inflammatory NO production as the principal bioactivity of the EtOAc extract of the newly isolated endophytic *F. equiseti* TU-64 from *P. maritimum*. The extract inhibited NO production in LPS-stimulated RAW264.7 macro-phages at concentrations below its overall cytotoxicity IC_50_, while complementary antioxidant and cholinesterase screens showed comparatively modest or very weak effects. LC–MS/MS tentatively annotated 31 metabolites, and iNOS docking prioritized several candidates, including fusarioxazin, for future investigation. Because the assays were conducted with a crude extract and the metabolite identities and docking predictions remain to be experimentally confirmed, no activity can yet be assigned to an individual compound. Bioactivity-guided fractionation, structural confirmation, and direct mechanistic validation are the essential next steps. Nevertheless, further studies are required to isolate and characterize the individual active compounds, elucidate their mechanisms of action, and evaluate their pharmacological efficacy and safety through in vivo investigations.

## Figures and Tables

**Table 1:** Total phenolic and alkaloid content and DPPH antioxidative activity (IC_50_) of EtOAc extract obtained from *F. equiseti*.

Extract	Total Phenolics (mg GAE/g)	Total Alkaloids (mg AE/g)	DPPH IC_50_ (mg/mL)
*F. equiseti*	29.31 ± 0.30	8.43 ± 0.29	2.18 ± 0.17
BHT	-	-	0.15 ± 0.01

Data are shown as mean values with corresponding ±SD from three replicates.

**Table 2:** Cytotoxic activity against RAW264.7 cells (IC_50_) and brine shrimp lethality (LC_50_) of the EtOAc extract of the endophytic fungus *F. equiseti* TU-64.

Extract	RAW264.7 IC_50_ (μg/mL)	*Artemia salina* LC_50_ (mg/mL)
*F. equiseti*	49.95 ± 0.45	1.86 ± 0.12
Quercetin	529.21 ± 10.77	NT

NT: Not tested. Data are shown as mean values with corresponding ±SD from three replicates.

**Table 3:** Various compounds tentatively characterized via LC-MS/MS analysis for *F. equiseti* extract.

No.	Compound	Chemical Formula	RT	Mass	[M + H]^+^	MS^2^ Fragments	Source	Ref.
1	Fusariumindole C	C_18_H_15_NO_4_	2.05	309.32	310.07	264, 162, 144, 121, 105, 103	*Fusarium* sp. L1	[48]
2	Acuminatopyrone	C_11_H_11_NO_3_	2.90	205.21	206.08	190, 188, 178, 176, 160, 148, 146, 125	*F. acuminatum* C.M.I. 35098	[49]
3	Astropaquinone D	C_17_H_18_O_5_	3.10	302.33	303.10	285, 271, 257, 243, 215, 205, 179, 125	*F. napiforme*	[50]
4	O-ethylhydroxydihydrofusarubin	C_17_H_20_O_8_	3.62	352.34	353.12	335, 307, 293, 289, 253, 249	*F. solani*	[51]
5	Fusaric acid	C_10_H_13_NO_2_	3.82	179.22	180.08	162, 138, 120	*F. oxysporum*	[52]
6	Fusarin H	C_9_H_14_O_4_	5.20	186.21	187.02	155, 141, 137, 127, 123, 111, 109	*F. solani* 7227	[53]
7	(-)-3a-Hydroxyfuroindoline	C_10_H_11_NO_2_	6.61	177.20	178.07	160, 150, 142, 134, 128	*F. incarnatum* (HKI00504)	[54]
8	Fusaric acid methyl ester	C_11_H_15_NO_2_	7.03	193.25	194.10	162, 132, 120	*F. nygamai*	[55]
9	9,10-dehydrofusaric acid methyl ester	C_11_H_13_NO_2_	7.56	191.23	192.08	174, 160, 150, 132, 118, 105	*F. nygamai*	[55]
10	5-hydroxy-3-methoxydihydrofusarubin A	C_16_H_20_O_7_	7.79	324.33	325.14	293, 275, 265, 261, 251, 239, 211, 197	*Fusarium* sp. BCC14842	[56]
11	Fusagunolic A	C_9_H_8_O_5_	8.11	196.16	197.06	179, 169, 137, 123, 109	*F. guttiforme*	[57]
12	Fusarpyrone A	C_10_H_12_O_3_	8.33	180.20	181.06	161, 149, 135, 121, 119, 115, 105	*F. solani* PSU-RSPG37	[58]
13	Fusarester A	C_14_H_20_O_3_	8.54	236.31	237.10	219, 205, 185, 181, 145, 139, 125	*Fusarium* sp. Hungcl	[59]
14	Karimunone A	C_17_H_12_O_8_	8.80	344.28	345.03	327, 309, 299, 273, 245, 233, 219	*Fusarium* sp. KJMT.FP.4.3	[60]
15	Fungerin	C_13_H_18_N_2_O_2_	9.13	234.30	235.14	217, 193,179, 161, 145, 135	*Fusarium* sp.	[61]
16	7-iso-cucurbic acid	C_12_H_20_O_3_	9.71	212.29	213.14	195, 177, 157, 153, 135, 121, 109	*F. oxysporum* f. sp. *matthiolae*	[62]
17	6-Hydroxy-astropaquinone B	C_18_H_20_O_6_	9.84	332.35	333.12	315, 301, 287, 273, 259, 245, 231	*F. napiforme*	[50]
18	Neomangicol C	C_25_H_36_O_5_	10.60	416.56	417.10	399, 381, 371, 293, 267, 251, 211	*Fusarium* sp.	[63]
19	(-)-byssochlamic acid imide	C_18_H_21_NO_5_	11.11	331.37	332.10	314, 304, 285, 213	*F. graminearum* K38 and E33	[64]
20	Wortmannin	C_23_H_24_O_8_	11.90	428.44	429.10	411, 397, 369, 351, 349, 341, 291, 259, 186	*Penicillium wortmannii*	[65]
21	Decalintetracid A	C_22_H_29_NO_4_	14.43	371.48	372.12	354, 344, 298, 264, 229, 203, 189	*F. equiseti* D39	[66]
22	Fusarielin H	C_25_H_38_O_3_	16.09	386.58	387.21	309, 263, 241, 233, 201, 187, 175, 153, 135	*F. graminearum*	[67]
23	Fusaritricin A/B	C_11_H_16_O_4_	16.20	212.25	213.10	197, 185, 179, 169, 155, 141	*F. tricinctum*	[68]
24	Fusarithioamide B	C_20_H_29_N_5_O_6_S	16.23	467.55	468.18	450, 391, 344	*F. chlamydosporium*	[69]
25	Fusarilactone A	C_12_H_16_O_5_	16.46	240.26	241.07	223, 213, 195, 183, 165, 155	*Fusarium* sp. #001	[70]
26	5′-Hydroxyzearalenol	C_18_H_24_O_6_	16.70	336.38	337.17	319, 245	*Fusarium* sp. 05ABR26	[71]
27	Equisetin	C_22_H_31_NO_4_	17.53	373.49	374.27	356, 330, 312, 252, 203, 175	*F. equiseti*	[72]
28	2,2-dimethyl-5-amino-6-(4′-hydroxylbutyryl)-4-chromone	C_15_H_19_NO_4_	19.24	277.32	278.10	260 246, 236, 234, 222, 216, 194	*F. equiseti*	[73]
29	Fujikurin D	C_12_H_18_O_4_	21.25	226.27	227.13	171, 143, 129, 123, 109	*F. fujikuroi*	[74]
30	Ac-r-N-DMAT	C_18_H_22_N_2_O_3_	24.71	314.39	315.17	297, 255, 227, 201	*F. fujikuroi*	[75]
31	Fusarioxazin	C_20_H_19_NO_4_	25.48	337.38	338.14	310, 296, 282, 264, 256, 240, 225	*F. oxysporum*	[76]

**Table 4:** Binding affinity values (kcal/mol) of tentatively identified compounds with iNOS (PDB ID: 3E7G).

No.	Ligand	Binding Affinity (kcal/mol)	No.	Ligand	Binding Affinity (kcal/mol)
1	Fusariumindole C	−6.7	17	6-Hydroxy-astropaquinon B	−7.8
2	Acuminatopyrone	−6.4	18	Neomangicol C	−8.3
3	Astropaquinone D	−7.7	19	(-)-byssochlamic acid imide	−8.2
4	O-ethylhydroxydihydrofusarubin	−7.9	20	Wortmannin	−8.0
5	Fusaric acid	−5.6	21	Decalintetracid A	−8.2
6	Fusarin H	−5.3	22	Fusarielin H	−8.3
7	(-)-3a-hydroxyfuroindoline	−6.2	23	Fusaritricin A/B	−6.5
8	Fusaric acid methyl ester	−5.4	24	Fusarithioamide B	−7.4
9	9,10-dehydrofusaric acid methyl ester	−5.6	25	Fusarilactone A	−6.4
10	5-hydroxy-3-methoxydihydrofusarubin A	−7.1	26	5′-Hydroxyzearalenol	−8.5
11	Fusagunolic A	−5.9	27	Equisetin	−8.0
12	Fusarpyrone A	−5.7	28	2,2-dimethyl-5-amino-6-(4′-hydroxylbutyryl)-4-chromone	−6.5
13	Fusarester A	−6.3	29	Fujikurin D	−6.2
14	Karimunone A	−8.5	30	Ac-r-N-DMAT	−7.0
15	Fungerin	−6.2	31	Fusarioxazin	−8.8
16	7-iso-cucurbic acid	−6.0		AR-C95791 complexed ligand (Reference)	−6.7

## Data Availability

Data of the measurement results are available from the authors.

## Supplementary Materials

The following supporting information can be downloaded at https://www.mdpi.com/article/10.3390/microorganisms14081773/s1. Table S1: Cholinesterase inhibitory activity of the *F. equiseti* TU-64 EtOAc extract. Reference [97] is cited in the supplementary materials.
