# Supplementary material for: Evaluation of the Biological Potential of an Endophytic Fusarium equiseti from Pancratium maritimum Supported by Metabolic Profiling and Molecular Docking
Source: Microorganisms. 2026 Aug 12;14(8):1773. doi: 10.3390/microorganisms14081773 (PMC13515202; doi:10.3390/microorganisms14081773)
Supplement: Supplementary file 1 [file microorganisms-14-01773-s001.zip › microorganisms-4459540-supplementary.pdf]

microorganisms

Supplementary

# Evaluation of the Biological Potential of an Endophytic *Fusarium equiseti* from *Pancratium maritimum* Supported by Metabolic Profiling and Molecular Docking

**Amal A. Al Mousa [1,*,†], Mohamed E. Abouelela [2,†], Reda A. Abdelhamid [3], Hassan Mohamed [4,5], Mohammed M. M. Abdelrahem [4], Mohamed F. Awad [4], Shurouq A. Fahmy [6], Huda A. Aljhne [1], Nageh F. Abo-Dahab [4] and Abdallah M. A. Hassane [4,*]**

[1] Department of Botany and Microbiology, College of Science, King Saud University, P.O. Box 145111, Riyadh ZIP 4545, Saudi Arabia; 445205534@student.ksu.edu.sa

[2] Department of Pharmacognosy, Faculty of Pharmacy (Boys), Al-Azhar University, Cairo 11884, Egypt; m_abouelela@azhar.edu.eg

[3] Department of Pharmacognosy, Faculty of Pharmacy (Boys), Al-Azhar University, Assiut Branch, Assiut 71524, Egypt; reda.ahmed@azhar.edu.eg

[4] Department of Botany and Microbiology, Faculty of Science, Al-Azhar University, Assiut Branch, Assiut 71524, Egypt; hassanmohamed85@azhar.edu.eg (H.M.); mohammedabdel-rahem.42@azhar.edu.eg (M.M.M.A.); m.fadl@tu.edu.sa (M.F.A.); abodahabn@azhar.edu.eg (N.F.A.-D.)

[5] Colin Ratledge Center for Microbial Lipids, School of Agricultural Engineering and Food Science, Shandong University of Technology, Zibo 255000, China

[6] Ministry of Health and Populations, Cairo P.O. Box 11516, Egypt; shorouk.ali@msa.edu.eg

* Correspondence: aalmosa@ksu.edu.sa (A.A.A.M.); abdallahhassane@azhar.edu.eg (A.M.A.H.); Tel.: +966-505389419 (A.A.A.M.); +20-1122990125 (A.M.A.H.)

† These authors contributed equally to this work.

**Evaluation of the Biological Potential of an Endophytic *Fusarium equiseti* from *Pancratium maritimum* Supported by Metabolic Profiling and Molecular Docking**

*Acetylcholinesterase and butyrylcholinesterase inhibition assay*

Cholinesterase inhibition was evaluated as part of the initial pharmacological screening. Because the activity was very weak and is not a central finding of the study, these data are provided here as supplementary information.

**Materials and method**

The fungal extract was tested at final concentrations ranging from 50 to 500 μg/mL. Stock solutions were initially prepared by dissolving the extract in dimethyl sulfoxide (DMSO) (MilliporeSigma, St. Louis, MO, USA) and subsequently diluted with distilled water to obtain the desired test concentrations. The reference inhibitor, Donepezil, was prepared in methanol at final concentrations of 1, 0.1, 0.01, 0.001, and 0.0001 μM, corresponding to 0.416, 0.0416, 0.00416, 0.000416, and 0.0000416 μg/mL, respectively. The inhibitory activity was evaluated using acetylcholinesterase and butyrylcholinesterase, along with their respective substrate's acetylthiocholine iodide and butyrylthiocholine iodide, and 5,5′-Dithiobis-(2-nitrobenzoic acid) DTNB as the chromogenic reagent. The assay was carried out following the procedures described by Osman et al. [97] with slight modifications. In brief, 10 μL of 0.4 mM DTNB prepared in 100 mM Tris–HCl buffer (pH 7.5) was added to each well of a 96-well microplate (Greiner Bio-One, Kremsmünster, Austria). Subsequently, 20 μL of enzyme solution (acetylcholinesterase or butyrylcholinesterase (Sigma-Aldrich, Germany) was added. The enzyme solution was prepared in 50 mM Tris–HCl buffer (pH 7.5) supplemented with 0.1% bovine serum albumin (BSA) to achieve a final enzyme concentration of 0.02 U/mL. Thereafter, 20 μL of the fungal extract was added to each well, and the reaction volume was adjusted by adding 140 μL of 100 mM Tris–HCl buffer (pH 7.5). The reaction mixture was incubated at room temperature for 15 min. Subsequently, 10 μL of substrate solution containing either 0.4 mM acetylthiocholine iodide or 0.4 mM butyrylthiocholine iodide (Merck, Rahway, NJ, USA) was added to initiate the enzymatic reaction. The microplate was then incubated in the dark at room temperature for an additional 20 min. Finally, the absorbance was measured at 412 nm using a microplate reader (Tecan, Männedorf, Switzerland), and the $IC_{50}$ values were calculated relative to the control.

**Results**

The EtOAc extract of F. equiseti TU-64 showed very weak cholinesterase inhibitory activity, with $IC_{50}$ values of 380.30 ± 22.87 μg/mL against acetylcholinesterase (AChE) and 400.60 ± 9.77 μg/mL against butyrylcholinesterase (BChE). These values indicate that the extract is not a promising target for cholinesterases inhibition.

**Table S1.** Cholinesterase inhibitory activity of the F. equiseti TU-64 EtOAc extract.

| Sample | AChE $IC_{50}$ (μg/mL) | BChE $IC_{50}$ (μg/mL) |
|---|---|---|
| F. equiseti TU-64 EtOAc extract | 380.30 ± 22.87 | 400.60 ± 9.77 |

Values are mean ± SD.

**Reference**

97. Osman, H.; Kumar, R.S.; Basiri, A.; Murugaiyah, V. Ionic liquid mediated synthesis of mono-and bis-spirooxindole-hexahydropyrrolidines as cholinesterase inhibitors and their molecular docking studies. *Bioorg. Med. Chem.* **2014**, 22(4), 1318-1328. https://doi.org/10.1016/j.bmc.2014.01.002
